# Homogenisation of Biocultural Diversity: Plant Ethnomedicine and Its Diachronic Change in Setomaa and Võromaa, Estonia, in the Last Century

**DOI:** 10.3390/biology11020192

**Published:** 2022-01-26

**Authors:** Renata Sõukand, Raivo Kalle, Andrea Pieroni

**Affiliations:** 1 Department of Environmental Sciences, Informatics and Statistics, Ca’ Foscari University of Venice, Via Torino 155, Mestre, 30172 Venice, Italy; renata.soukand@unive.it; 2 University of Gastronomic Sciences, Piazza Vittorio Emanuele II 9, 12042 Pollenzo, Italy; a.pieroni@unisg.it; 3 Medical Analysis Department, Tishk International University, 100 Meter Street & Mosul Road, Erbil 44001, Iraq

**Keywords:** biological and cultural diversity, biocultural erosion, ethnomedicine, ethnobotany, Europe, Estonia, Seto

## Abstract

**Simple Summary:**

To understand how healing practices change over time, it is important to recognize the role and extent of external factors affecting the diversity of uses. Our exemplary case study is part of a larger project studying the influence of centralization on the use of medicinal plants. We examined the current and past plant use of two small communities that reside on the border with Russia and speak two dialects of Estonian, namely Seto and Võro. Our results show that within the lifetime of the people we interviewed, many earlier known uses were abandoned and new uses were strongly influenced by knowledge disseminated through centralized channels. Many such uses have also been recorded in geographically distant regions that once belonged to the Soviet Union. This demonstrates the homogenizing influence of centralized knowledge distribution, which has eroded place-based biocultural diversity. In order to secure the survival of knowledge on how to use locally grown plants, we suggest that more attention on the regional level needs to be given to preserving and supporting the distribution of such place-specific knowledge.

**Abstract:**

When studying the evolution of the use of medicinal plants, it is important to identify what role, and to what extent, external factors and local biocultural diversities play in shaping cultural changes. We chose as a case study, which forms part of a larger project, a religiously and linguistically distinct group, the Seto, and compared their current and past plant use with that of the surrounding Võro. Ethnobotanical fieldwork was conducted in the summers of 2018 and 2019. Current uses of plants constituted 34% of the total registered use reports and 41% of those were used to treat general diseases or used as prophylactics. In total, the medicinal use of 86 taxa was recorded, and of these 48 were prevalent. Strong erosion (the abandonment of 43, mainly wild taxa used historically) and valorisation of the uses shared with neighboring as well as distant regions once part of the Soviet Union, were evident, signalling the potential influence of the centralised distribution of knowledge. The results clearly show that the plant medicine-related biocultural diversities of the two groups have been considerably homogenised, eroded and influenced by the knowledge spread through various means during the Soviet era and over the last 30 years.

## 1. Introduction

The ethnomedicinal use of plants has been a popular research domain in Europe for the last three decades, with the number of references steadily growing in the last several years. A large majority of these European studies (2/3) were conducted in the Mediterranean region (Italy and Spain) and Turkey [1]. Since 2016, the number of ethnopharmacological papers published on post-socialist and post-communist countries has increased remarkably, as the area presents a good model to study the effects of centralisation (of medical, education, and political systems) and its potential influence on the evolution of the use of medicinal plants. The use of medicinal plants was also highly diverse in response to the COVID-19 pandemic [2] and therefore deserves future investigation.

The practice of medicinal plant use is constantly evolving, as old uses are abandoned and new ones accepted from among both local and cultivated flora [3,4]. Yet, as medicinal plant use is not a basic need for survival, as is true for wild food during food shortages, it is considered more restricted by cultural boundaries [5]. We also see this in the case of migrant ethnobotany, where the medicinal use of plants is rather culture-specific and even if the plant is not growing in the new location similar substitutes are sought [6]. Menendez-Baceta et al. [7] demonstrated that in four Basque regions of Spain which share the same vegetation, four clearly discernible medicinal floras have appeared.

The religion and language spoken by local communities influence the use of wild food plants, which is a domain much more stable and shared within a community [8,9]. We should thus expect higher diversity in medicinal plant use among religiously and linguistically diverse communities, even if their wild food use is not remarkably varied [10]. For this reason, we selected a group, which although part of a larger study, still deserves attention due to its linguistic and religious distinctiveness. We selected two neighbouring groups in Estonia, the Seto and Võro, which are closely related through linguistic ties (the Seto language, although sometimes considered a separate language, is viewed by linguists as a subdialect of the Võro dialect of the Estonian language), but which have two distinct religious systems: the Seto being Orthodox while the Võro are Lutheran. While Estonians have been considered the least religious people in Europe (according to a 2011 census 64% of Estonians are religiously unaffiliated), this does not apply to the Seto who, in the majority, actively attend the Orthodox Church. This provides a basis to assume that we will be able to evaluate the effect of approximately 45 years of Soviet centralisation (which in the sphere of medicine included the promotion of formally accepted medicinal plants, referred to as “officinal”) on the folk medical system of these two religiously distinct groups.

The aims of the current work were:

(a) To document and analyse current and past uses of medicinal plants.
(b) To conduct a cross-cultural comparison between the Seto and Võro.
(c) To conduct a diachronic comparison of the changes in plant use within the lifetime of one individual.
(d) To conduct a diachronic comparison with available historical data on Seto plant ethnomedicine.

We expected to see a variety of uses between the two linguistic groups given their diverse religious backgrounds, and yet at the same time we expected to see pan-Soviet elements in the ethnomedicine of the selected groups as a result of centralised medical services and plant use propagation.

## 2. Materials and Methods

### 2.1. The Research Region

Old-Võromaa (in Estonian: Võrumaa) and Setomaa (in Estonian: Setumaa) are shaped by the highest elevation in the Baltic states, the Haanja Upland (318 m), and one of the largest inland water bodies in Europe, Lake Peipus (3555 km^2^), which also currently serves as a natural border with the Russian Federation. Pastures, characteristic of Setomaa and Võromaa, were intensively grazed in the past, but now, due to the end of many agricultural and economic activities, are losing their species richness [11]. The region began to be depleted of ice about 14,000 years ago, and therefore the vegetation of south-eastern Estonia is one of the oldest in the country. The South-East Transition Road served as the route for the reintroduction of plants to the territory of present-day Estonian. Historically dominated by coniferous forests, this area has low-yielding sandy soils, pine forests and fertile areas with spruce forests, being one of the least species-rich vegetations in Estonia, which is continuing to disappear today. In the Estonian part of Setomaa, 149 of the 717 native species (including subspecies) disappeared from 1971 to 2005, with an additional 88 species becoming very rare [12] and continuing to disappear into the 21st century [13].

**Figure 1:**
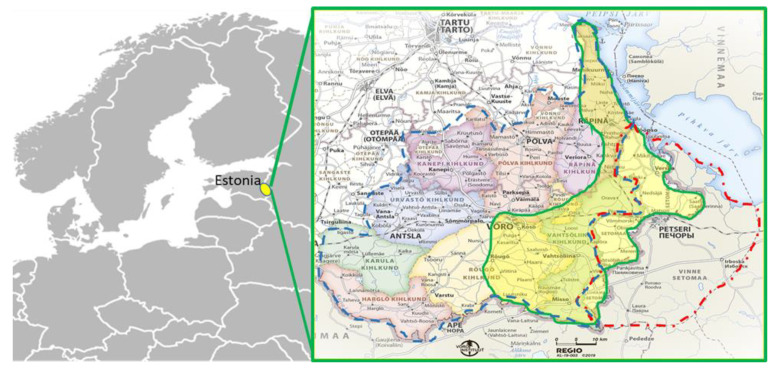
Left: location of the research area in Estonia. Right: the approximate study area (yellow) is surrounded by a green line. The administrative boundaries of Old-Võromaa (blue) and Setomaa (red) are also depicted. The place names are in the Võro dialect (Source: https://wi.ee/, accessed on 20 December 2021).

The research covered two historical areas: Old-Võromaa and Setomaa (Figure 1). Administrative boundaries of the region under study have repeatedly changed. In the ethnographic and folkloristic research, the division is based on the parishes of the late 19th century; our area of interest of Old-Võromaa belonged to Räpina, Vastseliina, Rõuge and Põlva parishes. While until the 1920s Estonians adhered to the legal norms of the Estonian or Livonian governorates, Setos belonged to Pskov Governorate, Pskov County, living mostly on lands belonging to Pechory Monastery. Setomaa as an administrative entity was established after the Estonian War of Independence in 1920 and existed until the end of World War II, when a large part of the territory of historical Setomaa was incorporated into the Russian SFSR. The fieldwork was conducted in the smaller area, which remained part of Estonia. According to the last administrative reform of 2017, the study area currently includes four official rural municipalities: Võru (10,738 inhabitants), Setomaa (3280 inhabitants), Rõuge (5427 inhabitants) and Võru town (11,859).

In Old-Võromaa, historically the Võro dialect was predominantly spoken, although many other dialects were also spoken in this territory. The whole of Setomaa has been historically inhabited by speakers of the Seto language, which is now considered a subdialect of the Võro dialect of Estonian. In the territory of Old-Võromaa, village and town schools that taught in Estonian were already established in the 19th century. The Seto, as they were then living in Pskov Governorate, had schools in which the language of instruction was Russian. Therefore, a limited number of Seto people could obtain a school education, and thus the Seto remained predominantly illiterate until the beginning of the 20th century. Literacy, along with the first surnames, were only obtained when the Seto were incorporated into the Republic of Estonia, which brought about rapid Estonianisation [14,15,16]. Now standard Estonian is the main language of communication in both regions and the dialects are preserved mainly as home-spoken languages, with only a few hours of instruction per week in the school curriculum. The regional newspapers “Setomaa” (published since 1995 in both Seto and Estonian, and since 2012 only in Seto—https://setomaa.kovtp.ee/ajaleht-setomaa, accessed on 20 December 2021) and “Umma leht” (published since 2000 in Võro—https://umaleht.ee/, accessed on 20 December 2021) and the journal “Peko Helü” (published since 2006) contribute to the development of vocabulary in the two dialects.

Small, unfertile farmlands in Setomaa and part of Old-Võromaa have resulted in the lowest standard of living in Estonia throughout history. This has to a certain extent persisted to the present day, which has caused intensified migration of young people to cities [14]. No large-scale industrial production has existed in the area, while small farms have been engaged in growing vegetables such as cucumbers, onions and strawberries [17,18,19]. These have been supplemented by trading and brokerage and small industries, which to today have mostly produced small-scale crafts, especially ceramics. In the 19th century, overpopulation and poverty resulted in several mass emigration waves to Siberia, where the land was given free of charge [20]. Since the start of the 1920s, there have been seasonal labour migrations to the inland of Estonia, and also to Latvia, especially to work in the large industrial enterprises of northern Estonia [21,22,23,24]. Migration within Setomaa intensified after the closure of Estonian-language schools during Soviet times in the areas that were assigned to Pechorsky District, culminating in 1997 after the full closure of the border [25].

At the end of the 19th century, there were still a large number of folk or witch doctors in Võru (historically: Wörro) county. They were feared, but people nevertheless went to them for help in times of illness [26]. By the beginning of the 20th century, however, in some regions of Võromaa, folk doctors had almost died out [27]. In addition to going to folk doctors, people in Võromaa and Setomaa visited medicinal stones, springs and trees, which were found everywhere in the region, to treat themselves. The most important ones, such as Miikse Jaanikivi and Silmaallikas, the Võhandu River (which is called Pühajõgi (translated as “holy river”) near the town of Võru), and the sacred oak of Pechory Monastery, were visited by people from all over the region. On the Republic of Estonia side of the border, former “natural spas” have now been given nature or heritage protection. It is important to note that in the 19th century there were no doctors in the rural areas of the Livonian Governorate (which also included Võru county), as they were located only in towns [28], e.g., Võru and the neighbouring governorate of Pechory. There were also rural hospitals in Võru (opened in 1827) and Pechory (c. 1890s) as well as pharmacies (opened in 1785 in Võru and in 1865 in Pechory); the opening of pharmacies particularly increased after the Estonian War of Independence [29].

After the occupation of Estonia by the USSR in the 1940s, the Estonian constitution of the USSR (based on the USSR constitution) came into force, which claimed to provide the right of all citizens to have free state health care and social services. Throughout the whole Soviet Union, the regime provided false psychological assurance of free medicinal care which was accepted by the population as its due [30]. The reality, however, was far from ideal and the actual accessibility and quality of medical care depended on the status and wealth of the individual and/or the region [31]. It was quite common for doctors to share with patients alternative treatment methods (including the use of medicinal plants) and to accept “gifts, tips, or preferential access to scarce consumer goods from their patients” [32].

The planned economy also had a strong influence on the collection of medicinal herbs, with pharmacists and foresters being obliged (by national standards) to collect medicinal herbs and give them to the state. Pharmacies, in turn, directed children, through schools, to collect medicinal plants. With the re-establishment of the Estonian Republic in 1991, compulsory health insurance was created for working people. A person who does not have it (e.g., unemployed, homemaker, etc.) has to paid for specialist medical care themself or purchase insurance. Pregnant women, children up to 18 years of age, pensioners and people with disabilities are guaranteed state health insurance [33]. Estonia has the highest level of unmet medical needs (especially in dental care) in the EU and the longest waiting time to see a doctor, which depends little on the income of the patient.

### 2.2. Fieldwork

Fieldwork was conducted in the summers of 2018 and 2019 and covered historical Old-Võromaa and the part of historical Setomaa, which is now the territory of Estonia. The sample was designed to solely include local people, even if some of them had lived or worked elsewhere in Estonia for shorter or longer periods. The youngest interviewee was 35 years old, while the oldest was 93 years old. Of the 71 interviewed people, 35 were individuals living in the territory of Võromaa that did not consider themselves Setos (hereafter the Võro group), while the remaining 36 participants were Setos living in Setomaa (hereafter the Seto group). Although the gender division was in favour of females (31 males and 40 females) due to their greater availability for interviews and their longer life-expectancy in Estonia (see also [34]), the gender distribution between the different groups was well balanced. The education level of the sample was consistent with the general education level of the older generation: 36% primary school, 25% high school, 24% university and 15% vocational training. The occupations carried out by the interviewees were quite diverse, and included jobs in the agricultural sector such as kolkhoz (collective farm) and sovkhoz (state farm) workers (farmers and farmer assistants, agronomists, herdsmen, milkmaids, beekeepers, tractor drivers, truck drivers, fishermen, zootechnicians), as well as forestry workers (foresters, hunters), clerical workers (accountants, civil servants, clerks) and many other professions (chefs, teachers, cleaners, border guards, construction workers, carpenters, car mechanics, etc.). Many of the interviewee had kept two or more different jobs, mainly due to the changes in employment opportunities that occurred after the fall of the Soviet Union and the closure of the kolkhozes and sovkhozes.

Oral consent was obtained after the study goals were explained to the interviewees, and with their permission we recorded the interviews for better processing. About 50% of the interviewees also provided written consent at the end of the interview. The study was approved by the Ethics Committee of Università Ca’ Foscari and strictly followed the ethical guidelines outlined by the International Society of Ethnobiology [35]. The semi-structured interviews lasted from 0.5 h to over 2 h covering food, medicinal and other uses of the plants.

The medicinal plant use section of the interview was conducted after the questions on wild food. It was particularly stressed that we were interested in all kinds of home medications, including cultivated and purchased plants. Initial “free listing” was attempted, but it was usually very short and rarely succeeded. After that, plant remedies used for different emic disease categories were asked about, following a mind-mapping system, starting with the head (headache, cold, ear and eye diseases, sore throat, etc.) and internal organ diseases (stomach, heart, lungs, kidneys, etc.), followed by systemic disorders (joint diseases, diabetes, allergies, cancer, immune system disorders), skin-related diseases and injuries (cuts, wounds, furuncles, rush), cultural-bound diseases (evil eye, nightmare, etc.) and lastly any other treated illnesses not yet named. If some of the plants included in free listings were not mentioned, the respondents’ attention was guided to those as well. We also asked interviewees to provide a date for the first and last use of the plant as precisely as possible to identify in which time period it was used. The interview was often followed by a walk around the house and surrounding forest, during which several new uses were pointed out by the participants.

Plants were identified on site and, wherever possible, herbarium specimens were collected. If offered, dry plant samples were also taken. Plant samples were stored at the Herbarium of Università Ca’ Foscari (UVV) bearing herbarium numbers SE001–SE141 (for herbarium specimens) and SEDR001–SEDR051 (for dried specimens). If the specimen was not available, the plant was identified on the basis of its popular name and full description of the plant and habitat. Some taxa (like *Hypericum*, *Betula*, *Mentha*) were identified only on the genus level as they are perceived in this way by people, even if voucher specimens were present for specific species of the genus, in order to avoid over-identification. The plant names follow Plants of the World Online [36] and the European Flora [37]; family assignments follow the Angiosperm Phylogeny Group IV [38].

### 2.3. Data Processing and Analysis

The field notes and, where possible, voice recordings were transcribed into a Word text document. The data on the plants used for medication were afterwards entered into an Excel spreadsheet based on Use Reports (UR). For this article we considered all plants used for self-medication, including wild, cultivated and purchased plants and their parts.

To understand changes in use within the lifetime of the interviewees, the results were analysed on the basis of the time the plants were used.

Past uses (no longer actively used):

− **Abandoned in childhood** and used by the informants’ parents, grandparents, and themselves as children (last used circa 1940s–1950s).
− **Abandoned in adulthood**—used in childhood and the use continued to some extent into adulthood (last used circa 1960s–1990s).
− **Temporarily** used in adulthood–learned as an adult and has been in use for some time following an acute need (used circa 1970s–2000s).

Current uses:

− The same use has been practiced **all the time** (used more or less constantly from childhood to the present).
− **New** uses—learned within recent years (since circa 2000, but mainly the last 5 years).

The uses were also compared group-wise.

We compared taxa recorded within the two communities and with historical sources to evaluate their ethno-medicinal distance by calculating Jaccard Similarity Indices (JI) following the methodology of González-Tejero et al. [39]:

JI = (C/(A + B − C)) × 100,

where A represents the number of taxa in sample A, B is the number of taxa in sample B and C is the number of taxa common to A and B.

Venn diagrams were created using the tool freely published by the Bioinformatics & Evolutionary Genomics laboratory at the University of Ghent (http://bioinformatics.psb.ugent.be/webtools/Venn/, accessed on 12 August 2021). Proportional diagrams were created using the PAST Toolkit Venn diagram plotter software program (https://omics.pnl.gov/software/venn-diagram-plotter accessed on 26 October 2021).

The results of the current study were compared with data obtained from historical folklore collections reflecting the use of plants documented in historical Setomaa and its surroundings (Vastseliina and Räpina parishes) from 1928 to 1942, mainly from the collection of the first Estonian ethnobotanist, Gustav Vilbaste (1885–1967), which was obtained with the help of school children [40]. Although there are a few earlier and later texts concerning the use of plants in Setomaa and its surroundings, they were not considered for the comparison as they are random in nature and did not employ the same collection methodology. The information provided in later texts (starting from the 1960s) might have already been influenced by Soviet centralisation.

For comparative purposes, emic disease names were correlated with International Classification of Primary Care, 2nd edition (ICPC-2, Updated March 2003) medicinal categories (hereafter etic disease categories). In the historical data, there were some emic disease names that were not correlatable with ICPC-2 classifications (such as evil eye or nightmare) and, therefore, an additional category of culture-bound diseases was adopted.

In order to discern the sources of homogenisation, we conducted a more focused comparison of taxa, which were named in at least three UR in this study (but not mentioned in more than 2 UR in historical sources), with studies carried out using a similar methodology within the territory of the former Soviet Union [41,42,43,44,45,46] and a few studies based on historical sources of plant use in Estonia territory [47,48,49].

## 3. Results

We recorded the use of 86 taxa belonging to 43 families (Table 1). The most represented families were Asteraceae and Rosaceae (both with 11 taxa), followed by Lamiaceae and Ericaceae (seven and six taxa, respectively). The most used families based on UR are Asteraceae (148 UR) and Ericaceae (70 UR), followed by Plantaginaceae (49 UR), Rosaceae (47 UR) and Amaryllidaceae (44 UR). Twenty-four taxa were named by one person and 12 by two people.

More than half of the taxa (45) were collected from the wild. Some wild taxa were also cultivated for medicinal use (e.g., *Origanum vulgare*) or for food, and then used for medicine (e.g., *Ribes nigrum*, *Rubus idaeus*), or purchased (e.g., *Valeriana officinalis*). Ready-made preparations of cultivated herbs were also purchased, such as *Sinapis alba* (patch and powder), *Coffea* (powder), *Syzygium aromaticum* (flower buds) and *Piper nigrum* (seeds). Fruits and vegetables were also used for treatment, including *Daucus carota* subsp. *sativus* (juice), *Prunus domestica* (juice) and home-made canned mixed vegetable salad (containing *Lycopersicon esculentum*, *Allium sativum*, *Armoracia rusticana*), which was eaten when there was an inflammation in the body or during a period of viral illness. Notably, cultivated *Ribes nigrum* was mainly used, while wild *Rubus idaeus* was preferred as medicine, likely because the availability of *Ribes nigrum* in the wild was rather limited while *Rubus ideaus* was routinely collected from the wild even when it was cultivated in the garden, as the wild form was considered “stronger medicine”. Another 32 taxa were solely cultivated, while two taxa (*Matricaria chamomilla* and *Capsicum annuum*) were both purchased and cultivated, mainly recently. Some taxa (e.g., *Zingiber officinale*) were only purchased and their use was highly influenced by popularisation.

The distribution of UR within the sample was not even: over 20 UR were reported by two people—56 and 25 UR respectively, 30 people reported from 10 to 19 UR, while one or two UR were reported by seven people. Each person used, on average, eight taxa. There were no statistically significant differences between genders or age groups in the number of taxa used. A local, newly established plant enthusiast and volunteer community healer living in Võru town named the largest number of plants (33), yet she primarily listed taxa whose uses were spread throughout the community, as only five taxa she named were not mentioned by anyone else. Those taxa, however, are not very exotic from the perspective of general plant use in Estonia (*Camellia sinensis*, *Chaenomeles japonica*, *Calluna vulgaris*, *Trifolium* pretense and *Solanum lycopersucyn*). She gathered her knowledge from different sources in Estonia, including the internet, and started healing with medicinal plants after retiring from an office job.

The most common disease categories treated with plants were general and unspecified diseases (35% of all uses), followed by skin (18%) and respiratory (16%) diseases (Table 2).

### 3.1. Cross-Cultural Analysis

**Figure 2:**
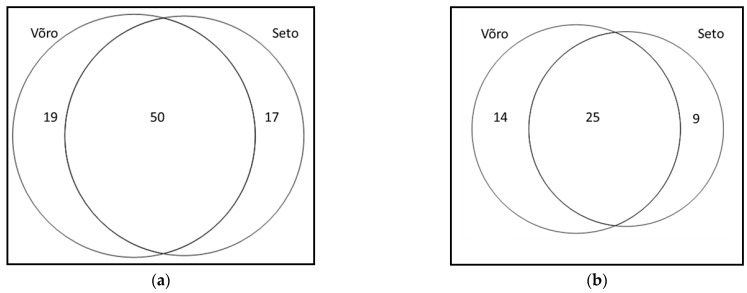
Overlap in the used plants based on cultural group: (**a**) all taxa, (**b**) taxa having at least three UR in either group.

Of the recorded 722 UR, 379 UR were recorded among the Võro and 343 among the Seto, resulting in a slightly higher mean use per person among the former group (10.83 for Võros vs. 9.27 for Setos). For all 86 taxa used, the difference between the groups was quite noticeable (JI = 58.74) (Figure 2), yet such diversity was caused mainly by singletons (18 in both groups). For the 48 taxa having three or more UR, the proportion of mutually used taxa was not very high: 25 taxa (JI = 52.08). However, the non-overlapping plants used solely by the Seto included the following four taxa: *Capsicum annuum*, *Thymus serpyllum*, *Echinacea purpurea* and *Nepeta cataria*. Both *Capsicum annuum* and *Thymus serpyllum* were historically very popular in Setomaa, while *Echinaceae purpurea* and *Nepeta cataria* represent recently introduced taxa.

The Võro exclusively used *Potentilla erecta*, *Viburnum opulus*, *Primula veris* and *Acorus calamus*. Remarkably, three of them (*Potentilla erecta*, *Viburnum opulus* and *Acorus calamus*) were historically well known and used, although moderately, in Setomaa. All the other plants were used for at least one UR in either group.

**Figure 3:**
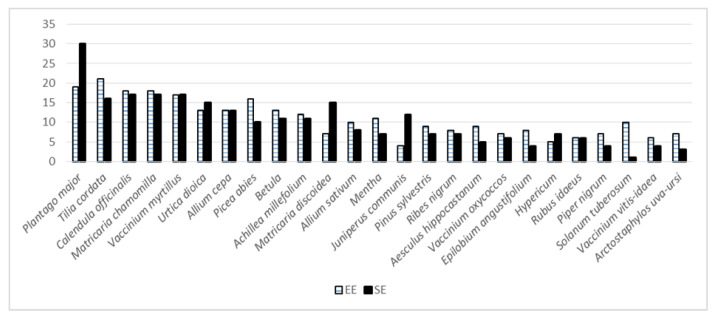
Distribution of UR of the 25 most popular plants in the two groups.

The distribution of UR between the 20 most used taxa was quite even, except for four taxa (Figure 3). The only taxon used almost exclusively by the Võro was *Solanum tuberosum*, although it is difficult to explain why, as the use of the steam from boiling potatoes to treat colds and bronchitis was widespread throughout all Estonia. The use of *Plantago major*, predominantly among Setos, owes it popularity, most likely, to its local name which refers to its use—*paiseleht* (translated as abscess leaf/boil leaf).

Given that the cross-cultural differences were minimal, further analysis did not differentiate on the level of language group.

### 3.2. Diachronic Analysis

**Figure 4:**
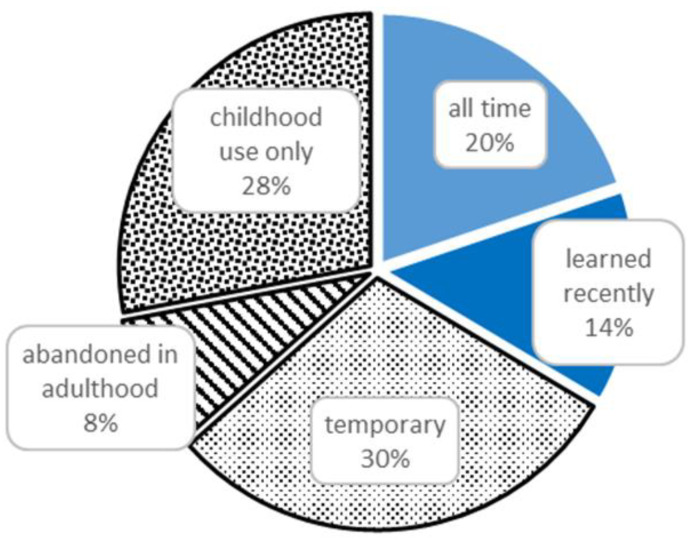
Proportion of UR used at different times in the lives of the interviewees.

**Figure 5:**
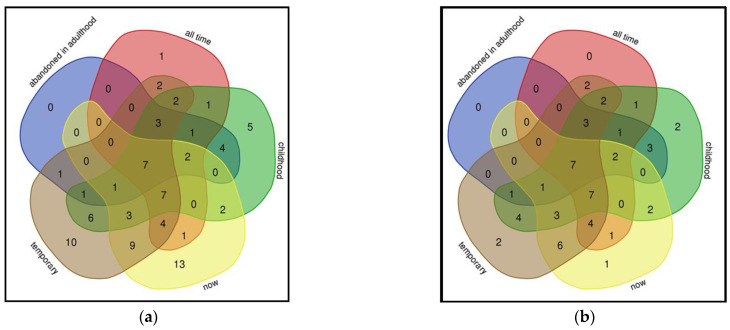
Overlap of plant uses in different periods of an individual’s life: (**a**) all taxa, (**b**) taxa used by at least three people.

A large portion (66%) of all UR represented past uses, while 34% reflected current uses (Figure 4). The diachronic division of the use of plants is highly individualistic and this shows the fluidity of the folk medicinal system; e.g., plants come and go when they are needed and do not remain in the repertoire all through life. While there are some specific plants used only now or temporarily in adulthood, the majority of these have fewer than three UR (Figure 5). For example, including singletons, there are 13 taxa that have come into use recently, yet only one of them, *Echinacea purpurea*, was mentioned in four use records and, notably, for two of them the exact use was not specified (*rohuks*, “just for medicine”). The number of all taxa used temporarily is about the same, yet only two of them, *Viburnum opulus* and *Thymus pulegioides*, are used more often. However, the list of taxa used in both diachronic domains (temporarily and now, e.g., the uses acquired during adulthood) is a bit longer: *Filipendula ulmaria*, *Rheum rhaponticum*, *Taraxacum officinale*, *Nepeta cataria*, *Epilobium angustifolium* and *Origanum vulgare*. The plants that were used only in childhood were rather less numerous (five), and of them *Avena sativa* and *Tanacetum vulgare* had at least three UR. There are also two iconic taxa whose use was abandoned in childhood by some individuals and picked up recently by others: *Potentilla erecta* and *Valeriana officinalis* were historically well known in the region and are now promoted by popular literature.

Seven taxa can be named as the core of an ever-changing medicinal plant system (*Achillea millefolium*, *Betula*, *Carum carvi*, *Hypericum, Matricaria chamomilla*, *Matricaria discoidea and Picea abies*), whose specific uses have, however, changed frequently through time.

**Figure 6:**
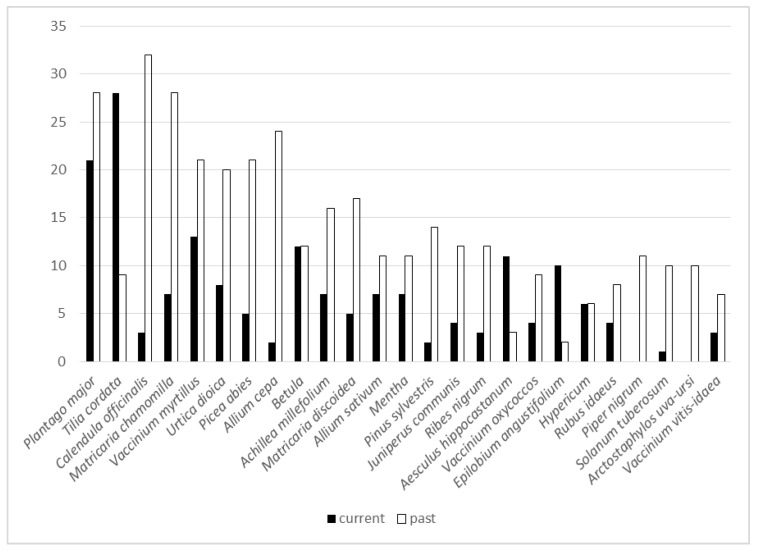
The past and current uses of the 25 most popular plants among the current study participants (UR).

Plant-wise, uses have changed remarkably. *Betula* is the only taxon whose intensity of use has remained the same, while the majority of the plants have lost their importance (Figure 6). *Arctostaphylos uva-ursi* was the only taxon among the most popular that was used solely in the past, mainly during childhood, but also temporarily in adulthood; the taxon was well known in historical sources and popularised in Soviet-era literature. The plants that have lost most of their importance included some that were intensively used in childhood but later abandoned, such as *Matricaria discoidea* that was often drunk in the absence of *Matricaria chamomilla*, which was temporarily used mainly in adulthood. *Allium cepa*, used mainly as a topical application on boils in childhood, was prepared by parents. The complexity of the preparation likely contributed to the decrease in its use, while *Plantago major*, also primarily applied topically, has retained its importance. *Pinus sylvestris* was mainly sourced for its resin for topical application on wounds in childhood, yet later the use changed, following the general trend, when the tea or tincture of the shoots became popular to treat respiratory diseases. However, this use did not last, as now the shoots are eaten fresh or preserved in sugar for the same application. The current use of *Picea abies* was slightly more diversified, yet past applications were mainly from childhood when the resin was used by itself or as a part of an ointment for healing wounds and abscesses. Similarly, the single current use of *Solanum tuberosum* differs in both application and mode of use (grated bulbs to treat eye inflammation) compared to its dominant childhood use (vapor of boiling bulbs against respiratory and general diseases) and temporary use in adulthood (starch to alleviate diarrhoea). *Calendula officinalis* was mainly used for some time in adulthood (promoted heavily in Soviet and post-Soviet time literature and commonly grown in gardens as an ornamental plant). *Piper nigurm* taken with vodka as a stomach ache remedy, frequently mentioned in historical sources [40], was mainly referred to as a temporary use in adulthood, as children were rarely introduced to alcohol-containing remedies.

Only three taxa out of the 25 most popular currently have greater importance. Of these, *Tilia cordata* was used all the time, predominately as a tea for respiratory and general (fever) diseases, while *Aesculus hippocastanum* was used in a tincture for topical application to treat muscular diseases, which has been popularised in recent literature. The only “new” popular plant is *Epilobium angustifolium*, whose small number of past uses were simply tried out in recent years, but now abandoned.

**Figure 7:**
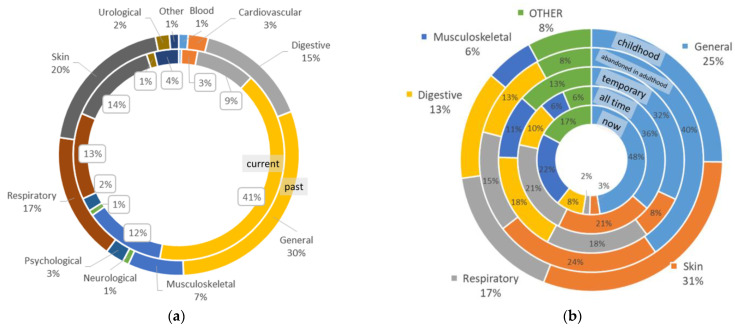
Division of UR among disease categories for past and current uses among the participating interviewees (**a**), and divided more precisely between all temporal categories (**b**).

The correspondence of emic disease names to etic disease categories (Table 2) shows the widespread treatment of some specific emic diseases such as colds (52 UR), wounds (45 UR), joint pain (33 UR) and fever (33 UR). However, the most widespread keyword used was “medicine” (71 UR), indicating that people know that a plant is medicinal and take it for this purpose, yet they do not remember the exact use, as less than one third (18 UR) of such uses refer to current uses. In general, the proportions of disease categories between past and current uses have changed, as the importance of plants for general and unidentified ailments and musculoskeletal diseases have increased, whereas their importance for digestive, skin and respiratory diseases have decreased during the lifetime of the interviewees (Figure 7).

The higher incidence of skin diseases in childhood is partly due to the fact that some skin diseases (e.g., verrucae, skin infections) were more common in children in the past due to inadequate hygiene and the fact that they are more likely to suffer accidental skin injuries. A higher proportion of joint diseases in older age is due to the development of chronic conditions (e.g., rheumatism). In the case of joint pain, new herbs are adopted very quickly and tested during such ailments. The reason for this is that the pain-relieving effects of plants diminish over time and new plants are therefore introduced. Compared to the past, people are also starting to place more emphasis on healthy foods, and this is boosting the abundance of plants used for vitamins and disease prevention.

### 3.3. Comparison of the Current Study with Folklore Collections in Setomaa and Bordering Regions from 1928–1942

**Figure 8:**
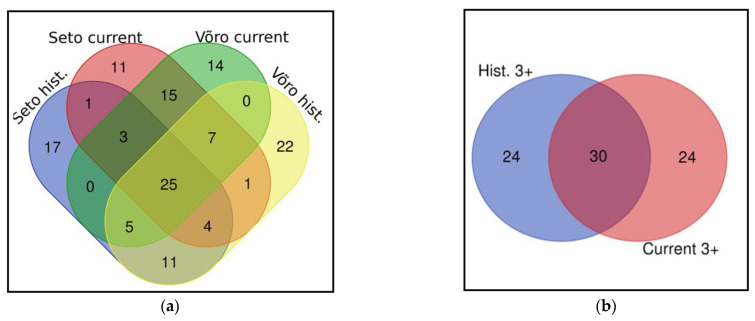
Venn diagram comparing plant taxa represented in folklore materials and the current study: (**a**) all plants in all four groups, and (**b**) plants having at least three UR in either study.

The number of plants recorded as used by the Seto in historical folklore is slightly greater than that used by Setos in the current study (Table 3). Yet, there is also similar overlap between the historical uses collected in areas neighbouring Setomaa and the uses among the Võro (Figure 8a).

However, when limiting the comparison to plants with three or more UR, the numbers are equivalent, leaving 54 taxa in the repertoire for both historical and current uses (JI = 38.46) (Figure 8b). For this, both studies were reflected as one region, given the high overlap of the commonly used taxa between the current Seto and Estonian groups and the impossibility of identifying the actual ethnic group of the person reporting the historical use (as there were also Estonians/Võros living in historical Setomaa and its vicinity in the 1930s).

**Figure 9:**
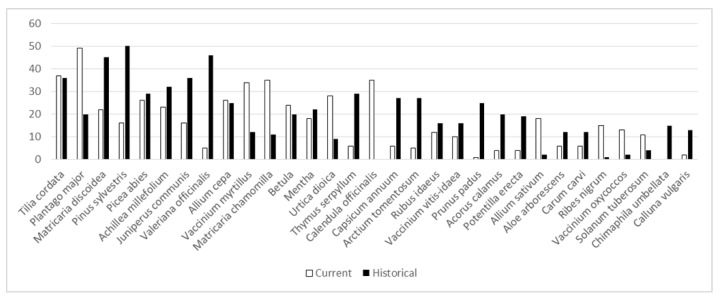
The most popular plants (with at least 15 UR) in the current study and based on historical records.

There is a clearer difference between the two studies for the most used taxa (those having at least 10 UR) (Figure 9). Among these, there is one taxon that was named only in the current study, namely *Calendula officinalis*, which started to become popular in the 1960s with its wider introduction into cultivation and promotion in popular literature. In addition, there are a few taxa rarely mentioned in historical sources that are very popular now. The most notable of these are *Plantago major, Matricaria chamomilla, Vaccinium myrtillus* and *Allium sativum*. The first taxon is mainly used for topical application on wounds and cuts, which may be the result of its widespread promotion in popular literature. *Matricaria chamomilla*, cultivated on some collective farms in the territory of the current Russian Federation, was historically largely unavailable, and thus its wild substitute *Matricaria discoidea* was predominantly used. However, the increased use of *Vaccinium myrtillus*, related mainly to digestive disorders, was also often referred to as a past use in the current study, while the cultivation of *Allium sativum* did not intensify before Soviet occupation. One taxon among the most used that was known only in the past is *Chimaphila umbellata* (endemic taxa), which was used for a wide variety of diseases and had folk names (*obijoon, obijon, opijon*, etc.) [40] obtained from the pharmacy drug “opium”.

**Figure 10:**
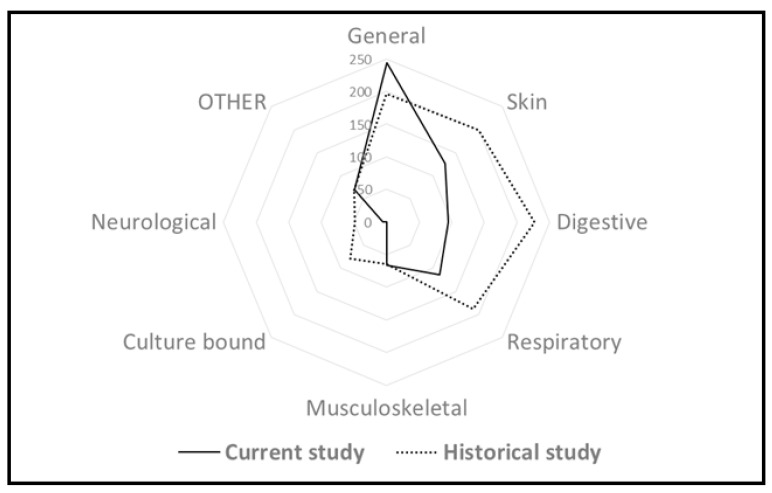
Comparison of the proportions of different etic disease categories in the current study and historical data.

There is a clear shift in the dominant diseases over time: skin, respiratory and digestive diseases dominate in the historical material, whereas currently the general disease category dominates (Figure 10). The prevalence of those disease categories in the historical data are due to the specific conditions of life in the first half of the 20th century. Poor sanitary conditions, cold houses and deficient or low-quality food were still well remembered in historical sources, even during the time of the first Estonian Republic, given that the collection of plant uses was directed mainly towards historical uses. In the current study, culture bound diseases (represented in the historical data) were completely absent as a result of the increase in education level and the availability of academic medical care in the population.

## 4. Discussion

### 4.1. Erosion and Evolution: Decrease in the Importance of Local Flora

The number of the used plants (86) recorded in the present study is comparable to the results obtained in earlier studies conducted in Ukraine with a smaller number of participants: 88 used plants by both Hutsuls living in Romania and Ukraine [46] and Romanians living in Ukraine. However, it is lower compared to the study among Hutsuls and Ukraine and Romania [41] and Romanians in Ukraine and Romania [42], both with comparable samples (111 and 108 taxa respectively) and much less compared to studies with larger samples: a study in Latgale (116) [43] and in Belarus (119) [44,45]. From the numbers of plants used, we cannot, however, detect remarkable erosion of knowledge, as the numbers are comparable to the recorded historical uses of plants among Setos and Võros. Yet, we need to keep in mind that the number obtained in the current study also includes many taxa that were used only earlier in the lifetime (past) of our interviewees and the number of plants currently used is as little as 59.

We can observe that among the taxa that have disappeared from use local species dominate (3/4 of the 40 taxa). When we attempt to correlate the no longer used taxa with the disappearance of diseases (such as culture-bound diseases that required the use of 11 taxa), we can observe that only one taxon (*Hylotelephium maximum*) was used solely for this disease category (having the name *kidsihain* and being applied in the case of a disease called *kidi* associated with wrist joint dislocation, most likely based on the similarity of the crunching sound produced in both cases). The name associated with a disease seems to be a strong driver for the plant use to erode from its historical use. For example, *Drosera rotundifolia*, named *huulhain* or one of its many variations (translating as lip hey), was used on the lips, and even though the name remains as the official name of the genus, not a single person recalled such a use. Other examples include *sammaspoolehain* (*Gnaphalium uliginosum*) used to treat a skin disease called *sammaspool* (probably some type of eczema) and *Hyoscyamus niger* referred to as *hambahain* (tooth herb) which was used to treat toothache. Yet, there are also exceptions such as *Achillea millefolium*, which is still present in use regardless of whether the disease-specific name is still in use, although its historically widespread name *verihain* (blood herb) was mentioned by only one person, as the majority of participants mentioned its current official name *raudrohi*. At the same time, the use of another taxon with a similar name (*verehain—Argentina anserina*) has been abandoned. Among the taxa whose use has been forgotten is *Urtica urens*, as its use as a medicinal plant was not recognised by Soviet medicine, while another species of *Urtica*, namely *Urtica dioica*, was widely popularised throughout the entire Soviet period. Quite characteristic of the present time are (wild) taxa purchased from pharmacies. The reason for this is either that these taxa are no longer found in nature (e.g., *Carum carvi* (seeds)) due to changes in landscape management, or simply that there is no longer the custom of picking plants from the wild (e.g., *Rosa* spp. (fruits)). The impact of pharmacies on the use of plants is seen especially in the introduction of cultivated species. A particular mention was made of learning about new uses of *Calendula officinalis* from pharmacists.

### 4.2. Changing Diseases and their Treatment

The Seto community has been well known throughout history for their reliance on sacred springs, trees and stones, as well as holy water and icons, in their medicinal treatments. There are 45 known cult stones from historical Setomaa alone, 29 of which were used for sacred, sacrificial or medicinal purposes. They were used by both the Seto people and Russians living in the area. Such a cultic use of stones in Setomaa was not an exception, but rather was common in all Baltic countries and north-west Russia [50]. Thus, such stones were also found in Old-Võromaa. In Setomaa, 28 trees are also known to have been considered sacred and/or used for healing, but historically there were many more. Again, the cultic use of trees is characteristic to the whole of the Baltic region and north-western Russia [51]. The tradition of springs being considered sacred and used for healing is similar in both Setomaa and Võromaa and is characteristic to the whole of north-western Russia, Scandinavia and the Baltics. However, the ratio of springs per unit area is almost twice as high in Setomaa (41 springs) as in Võromaa (43 springs). In addition, there are water bodies (rivers and streams) that are considered sacred and used for medicinal purposes [52]. Thus, the use of sacred spring water for medicinal purposes is more typical of the Seto. Therefore, for Setos (and to a lesser extent Võros), perhaps medicinal plants play a supportive role in disease treatment. Plants are used in addition to other alternative methods, such as additives to vodka for massaging painful joints, tempering in saunas, and washing the eyes with holy water in the case of orthodox Setos. Ether, which is used as an ointment and ingested for various ailments (mainly stomach ache), is also a popular alternative remedy among the Seto and Võro.

In the case of illnesses that have been diagnosed by a doctor, prescription medicines are taken, and herbal remedies are rarely involved in treating the illness. Trust in doctors was and still is high. The pharmacist is also an authority for most people, as the pharmacy is the first place one goes for help. In Estonian pharmacies, there has always been a very wide range of medicinal plants for sale, some of which are not used in folk medicine. Unlike during the Soviet era, in modern Estonian pharmacies wild plants are not purchased or collected, and cultivated plants are not grown in gardens. Pharmacies sell already packaged plants supplied internationally and by local providers who have the right to collect and package medicinal plants. The sale of medicinal plants is strictly regulated in present-day Estonia: medicinal products designated as officinal may only be sold in pharmacies and only by companies that comply with the requirements for the packaging of drugs (RTL 1998, 34, 185 https://www.riigiteataja.ee/akt/87274, accessed on 20 December 2021). There exists, however, so called “traditional use” plants (proven use for 30 years and at least 15 years in the EU), which may be used without the supervision of the person authorized to prescribe medicinal products, i.e., also outside pharmacies (RT I, 4 https://www.riigiteataja.ee/akt/104052016004, accessed on 20 December 2021).

In general, the emphasis on the use of medicinal plants has changed from treating diseases to preventing them. Plants are used in making healthy meals, as edible or drinkable prophylactics in case of viral diseases or in winter, as tonic teas or in spring for vitamins; thus, the prevalence of the general etic disease category among current uses, with the set of diseases very different from that common in the first half of the 20th century. Such a principal shift to diseases belonging to the general category, however, is due to societal changes, including changes in the epidemiological situation of the society (compulsory vaccination against tuberculosis, varicella, etc.), increased availability of easily accessible and relatively cheap medical preparations, the ever-increasing quality of healthcare and awareness of personal responsibility for one’s own well-being. For similar reasons the proportions of uses referring to the treatment of gastrointestinal and respiratory illnesses have decreased—food is more available and more diverse, while life conditions have much improved and even those working outside are better protected from severe weather conditions. It could also be that people better differentiate the causes of diseases and refer to a cold instead of its symptoms such as cough and running nose. Skin diseases are no longer one of the dominant use categories, which signals the gradual improvement of sanitary conditions in everyday life. Those uses from childhood may refer to accidental cuts often suffered by children. Here, we can make a comparison with Italy, where about the half of the medicinal ethnobotanical taxa recorded between 1960 and 1980 were used to treat skin problems and digestive issues. Subsequently and more recently these categories have decreased, and plant use has been much more linked to general health, the central nervous system, metabolic problems (reducing weight, etc.) and so on [53].

### 4.3. Resilience, Homogenisation and the Origin of New Plants in Ethnopharmacopoeia

During and after Sovietisation, overlap between the plants named in historical sources in Setomaa and that of neighbouring areas increased twofold for taxa used by at least three individuals (Table 3). However, the number of new and abandoned uses is comparable. Therefore, we should try understand the incorporation of new plants into local uses. Comparing the results of the current study with the historical data we can observe that among the taxa mentioned only in this study there is a wide range of so-called officinal medicinal plants whose use was promoted in the Soviet Union in some manner. Notably, 14 of the taxa were included in at least one of the pharmacopoeias of the Soviet Union.

Table 4 shows quite a distinctive pattern: the presence of 24 taxa mentioned by at least three people in the current study yet absent from the historical data gathered from medicinal plant studies conducted with a similar methodology in post-Soviet regions.

The greatest overlap in use (20 out of 24 taxa) is with a study conducted nearby in Latgale a few years ago [43]. The second largest overlap (16 of the same taxa out of 24 total) is with a study more geographically distant—the Ljuban region of Belarus [44,45]. The other three studies [41,42,46] conducted in Ukraine show an overlap of less than half of the taxa. Notably, only three taxa (*Allium sativum, Hypericum* and *Origanum vulgare*) have been named among the taxa widely used in Europe [1].

In general, we can divide the specific taxa into the following six groups.

(a) Those widely mentioned in early sources on Estonian ethnomedicine and therefore probably under-recorded due to earlier collection gaps or simply because they were little used regionally (*Filipendula ulmaria, Hypericum* (not differentiated at the species level), *Origanum vulgare, Alchemilla vulgaris, Tussilago farfara, Piper nigrum*) or not perceived as sourced from the plant (*Coffea*).
(b) Taxa used historically whose uses have changed considerably (*Epilobium angustifolium, Armoracia rusticana, Brassica oleracea*). The current use of *Epilobium angustifolium* is the result of intensive promotion of its use in recent literature and on the Internet (see also [54]).
(c) Taxa whose cultivation in home-gardens started (*Aronia melanocarpa, Calendula officinalis*) or intensified (*Allium sativum, Ribes nigrum)* during the Soviet period and whose uses were promoted in some manner through popular narratives (also *Aesculus hippocastanum*).
(d) Wild taxa popularized during the Soviet era in popular medicinal literature (*Rosa, Taraxacum officinale, Primula veris*).
(e) Taxa that have no overlap with any of the earlier studies. *Echinacea purpurea* and *Thymus pulegioides* are newly cultivated plants whose uses are of recent origin. *Thymus pulegioides* is used instead of *Thymus serpyllum* which is collected in the wild.
(f) The origin of the use of the remaining taxa is less clear, yet the uses are not very numerous and might have originated ad hoc, from random literature or media programs, or as a side-use of food plants also collected for sale (*Vaccinium oxycoccos*).

The resilience of the 30 taxa used in the past and recorded in the current study might also be due to homogenisation, as 25 of them also belong to officinal lists. The only exception among the wild taxa was *Matricaria discoidea*, not accepted as a medicinal plant in the Soviet system, while the remaining four (*Solanum tuberosum, Capsicum annuum, Avena sativa* and *Brassica oleracea*) are all cultivars used in both medical and food domains. The current findings show that the resilience of folk ethnomedical knowledge and practices can also be enhanced by the spread of literature popularising the use of local plants. This—apart from the specific former Soviet context—is surely an important factor to be kept in mind in other parts of the word when ethnobiologists debate policies aimed at fostering resilience and/or revitalisation of Local Ecological Knowledge.

### 4.4. School Procurement Campaigns as Vehicles for Introducing New Plants

The procurement of medicinal plants might be an important source of knowledge of medicinal plant use. During the Soviet era the collection of medicinal plants by schoolchildren was a compulsory task. Such plants can be divided into two groups. The first includes plants that were extensively used at home and collected for schools or pharmacies, e.g., *Matricaria chamomilla* (flowers, herb), *Matricaria discoidea* (herb), *Arctostaphylos uva-ursi* (leaves), *Vaccinium vitis-idaea* (leaves) and *Tilia cordata* (flowers). The second group contains plants (or specific plant parts) that were picked and dried for schools or pharmacies, but not used at home by some interviewees e.g., *Acorus calamus* (rhizome), *Betula* sp. (buds), *Hypericum* sp. (herb), *Plantago major* (leaves), *Valeriana officinalis* (roots) and *Achillea millefolium* (herb). For example, one Seto participant (F, born 1969) related, “And for school we also had to pick medicinal herbs. I was mainly there to pick *raudrohi* (*Achillea millefolium*), those flowers with short stems. At home, we tried the tea once, but it was bitter.” However, it is important to stress that the absence of the use of plants from the last group is very individual, as all of them have been mentioned, to a greater or lesser extent, as used now or in the earlier years of life of our interviewees.

What was actually collected by children depended more on the availability of the plants and the ease of collection. The following extract of an interview also highlights the diversity of uses, outside the medicinal domain, of the plants collected for pharmacies:

*When I was at school, it was necessary to bring herbs to school at the end of the summer break. Such was the obligation. We were actually not stupid then, we realised that picking linden blossoms was useless because they weighed nothing. Since my home was near the swamp, we picked the roots of the *palderjan* (Valeriana officinalis) from the edge of the swamp. We washed them clean, and cut the coarser roots lengthwise and dried them and carried them to school. They weighed considerably more. They were a lot of fun, because when they were drying the cats would go crazy and climb up on the roof to be near the *palderjan*. We didn’t use *palderjan* in our family. There were more plants on the list that we did not use at home, for example *naistepuna* (Hypericum *sp*.) and *kasekäsn* (Inonotus obliquus) come to mind, and we, the children, did not pick them for school assignments. We looked at what could be harvested more easily, that’s what we harvested, and we didn’t have any birch woods close by. There was also a plant on the school list that grows in water … *kalmus* (Acorus calamus), we picked the roots. And we dried them and took them to school, it was supposed to be some kind of medicinal herb, but again we didn’t use the roots at home. But we used to use the leaves of the *kalmus* at home to repel fleas. Then, on Saturday, when the house was all cleaned up and new straws were put in the sleeping bags, because we slept on the sleeping bags, then the calcareous leaves were brought in and cut into small pieces. These were then laid out on the floor and even on the beds. They made a very good smell. And then after they had been there for an hour or two, depending on how much time mother had, they would be swept up and taken out*.

(female, Estonian, born 1959)

The use of *Acorus calamus* to repel fleas was widespread in Estonia according to folklore sources [55], yet with the improvement of living conditions such a use has remained only a “sweet memory” of the past and many interviewees did not even remember why the leaves were actually used.

## 5. Conclusions

Our expectation of detecting a diversity of used plants was not met, and instead we found two linguistically (although on the dialect level) and religiously diverse groups using a predominantly very consistent list of plants that is shared between the two groups. An ongoing, and increasing, erosion of plant use is evident from the fact that the majority of uses are just recollections of the past and current practices are minimal. Almost half of the various historical uses, recorded rather sporadically, have vanished and are not even included in memories.

We also detected remarkable erosion of historical uses for both minority groups against the background of the diversity that existed at the beginning of the 20th century. At the same time, we can observe strong valorisation of the uses shared with neighbouring as well as distant regions. The obtained results clearly signal that the plant medicine-related biocultural diversities of the two study groups have been considerably homogenised and the entire diversity is covered mainly by plants mentioned by one or two people. The sources of homogenisation were academic medicine through medical personnel suggesting the use of a selected set of medicinal plants and, even more so, popular literature and TV (accessible to a larger portion of the population of the Soviet Union from the 1980s). The Soviet popular media were not free and self-directing as in non-Socialist Europe; but rather, as is characteristic of totalitarianism, specific information was spread in order to provide directions that would help to build a homogenised and obedient society. This allows us to draw the conclusion that frameworks created by totalitarianism destroy situated, place-based Local Ecological Knowledge, as by their nature they aim to make everything the same, restricting diversity.

## Figures and Tables

**Table 1:** List of plants and their uses.

Family	Taxa, Voucher n	Local Names	Plant Parts	Preparations	Applications	UR
Acoraceae	*Acorus calamus* L., SE054	kalmus	roots	tea	medicine	1
				tincture, topical application	painful areas	1
					toothache	3
Adoxaceae	*Viburnum opulus* L., SE051, SE068, SE119	lodjapuu, kalina	fruits	tea	cold	3
					high blood pressure	3
Amaryllidaceae	*Allium cepa* L.	sibul	bulbs	applied on the foot	cold	1
				baked, juice drunk	cough	1
				baked, topical application	abscesses	12
				boiled with milk (and honey)	cough	3
					sore throat	1
					cough	1
				crushed, put in the hair	hair loss	1
				eaten raw	cold	1
					inflammation	1
					internal parasites	1
					strengthen the blood	1
				topical application	abscesses	1
					sore throat	2
	*Allium sativum* L.	küüslauk, kurslak	bulbs	applied in the nose	cold	1
					stuffy nose	2
				eaten	disease prevention	1
					to strengthen the immune system	2
				eaten as salad	antiseptic	1
				eaten raw	gum disease	1
					to strengthen the blood	1
					good for every sickness	1
				infused in ether, rubbed into the hands	fever	1
				put in ear	earache	1
					headache	1
				tincture, drunk	inflammation	1
					internal parasites	1
					to strengthen the immune system	2
	*Allium ursinum* L.	karulauk	leaves	eaten fresh	vitamins in springtime	1
Apiaceae	*Carum carvi* L., SE031	köömen, köömned	seeds	bathe with decoction	foot pain	1
				tea	abdominal problems	1
					calming	1
					good for blood vessels	1
					good for the eyes	1
					medicine	1
	*Daucus carota* L. subsp. *sativus* (Hoffm.) Arcang.	porgand	roots	juice drunk	constipation in children	1
					headache	1
	*Petroselinum crispum* (Mill.) Fuss	petersell	leaves	tea	kidney disease	1
					medicine	1
Asphodelaceae	*Aloe arborescens* Mill.	aaloe	Leaves	juice drunk	medicine	1
				mixed with honey, eaten	to strengthen the immune system	1
				tea	cough	1
				topical application	burns	3
Asteraceae	*Achillea millefolium* L., SE077, SEDR009, SE062	raudrohi, verihain	aerial parts	tea	100 diseases	1
					against 9 diseases	1
					blood cleansing	2
					bronchitis	1
					cold	4
					cough	3
					medicine	7
				topical application	bleeding	1
			leaves	topical application	bleeding	2
					cuts	1
	*Arctium tomentosum* Mill., SE095	takjas	leaves	topical application	foot pain	1
					painful areas	3
					topic swelling	1
	*Calendula officinalis* L., SE029, SE105, SEDR001, SEDR024, SEDR038	saialill	flowers	chewed	headache	1
					sore throat	1
				ointment (with lard), topical application	abscesses	4
					inflammation	1
					psoriasis	1
					wounds	1
					inflammation	1
					insect bites	1
					scratches	1
					skin diseases	1
				tea	dental inflammation	1
					don’t know exactly	1
					indigestion	1
					inflammation	5
					inflammation of the stomach	1
					medicine	3
					sore throat	1
					stomach upset	1
				tea, gargled	oral inflammation	1
				tincture	gastric diseases	1
					sore throat	1
					to clean blood vessels	1
				tincture, topical application	joint pain	3
					pimples on face	1
	*Echinacea purpurea* (L.) Moench	siilikübar, punane päevakübar, päevakübar	flowers	tea	medicine	1
				tincture, drunk	medicine	1
				tincture, topical application	back pain	1
					joint pain	1
	*Helianthus tuberosus* L.	maapirn, maakartul	tubers	eaten	diabetes	1
	*Helichrysum arenarium* (L.) Moench, SEDR006	käokuld, pesmertnik	aerial parts	tea	liver cleansing	1
					medicine	1
	*Matricaria chamomilla* L., SE042, SE072, SE004, SE089, SEDR043	kummel, aed-kummel, apteegikummel, päriskummel, poekummel, teekumme, õigekummel	flowers	boiled, topical application	joint pain	1
				decocted, vapor inhaled	cough in children	2
				tea	cold	3
					cough	1
					dental inflammation	1
					good for everything	1
					had to drank after stomach surgery	1
					inflammation	5
					medicine	9
					several diseases	1
					stomach pain	1
					stomach pain in children	1
					stomach upset	1
					toothache	1
				tincture, topical application	wounds	2
				topical application	bee stings	1
					inflamed wounds	1
					painful eyes	1
					swollen legs	1
	*Matricaria discoidea* DC., SE017, SE066, SE043, SE005, SEDR002, SEDR019	kodukummel, murukummel, kummel, upinhain, tsäihain	aerial parts	steaming	bladder disease	1
				tea	cold	4
					fever	2
					good medicinal herb	4
					inflammation	4
					stomach gas in children	1
					stomach pain	1
					toothache	1
				infusion, topical application	inflammation	1
			flowers	drunk with honey	cold	1
	*Matricaria* sp.	kummel	aerial parts	tea	cough	2
	*Tanacetum vulgare* L., SE001, SE142	ussirohi, soolikarohi	flowers	eaten raw	prophylactic for worms	2
			flowers	tea	stomach pain	1
			seeds	dried	diarrhoea	1
				dried or raw	prophylactic for intestine worms	2
				eaten raw	stomach pain	2
	*Taraxacum officinale* F.H.Wigg. s.l., SE107	võilill	flowers	eaten in spring	strengthens the body	1
				tincture, drunk	medicine	1
				tincture, topical application	joint pain	1
			leaves	eaten fresh	vitamins in springtime	1
			leaves, flowers	eaten in salad	healthy	1
			roots	tincture	medicine	1
			stems	eaten raw	vitamins in springtime	2
					strengthens the liver	1
	*Tussilago farfara* L., SE055	paiseleht, ämmaleht	flowers	tea	expectorant	1
			leaves	topical application	painful areas	1
					topic swelling	2
Betulaceae	*Betula* sp., SE076, SE117	kask, kõiv, arukask	buds	tea	medicine	1
				tincture, drunk	gastric diseases	1
					to clean blood vessels	1
				tincture, topical application	pimples on face	1
			buds, catkins	tincture drunk	gastric diseases	1
				tincture, topical application	wounds	1
			charcoal	eaten	stomach ache	1
			twigs	applied honey on the body and whisked in sauna	back pain	1
					joint pain	2
				whisked in sauna	back pain	2
					good for health	2
					healthy	5
					joint pain	1
					muscle pain	2
				whisked with ash water	scabies	2
Boraginaceae	*Borago officinalis* L., SE010, SE084	kurgirohi	flowers	tincture	feeling bad	1
	*Symphytum asperum* Lepech., SE008	varemerohi	roots	tincture, topical application	joint pain	1
Brassicaceae	*Armoracia rusticana* G.Gaertn., B.Mey. & Scherb., SE014	mädarõigas	leaves	topical application	blood pressure	1
			roots	eaten as salad	antiseptic	1
				infused in vodka, topical application	inflammation	1
	*Brassica oleracea* L.	kapsas	leaves	sit on in sauna	healthy	1
				topical application	knee pain	4
					painful legs	2
					painful areas	1
				water from fermentation drunk	good for the stomach	1
	*Sinapis alba* L.	sinep	seeds	patches on chest and back	cough	4
				footbath	cold	2
Cannabaceae	*Humulus lupulus* L., SE028	humal	aerial parts	tea	good sleep	1
Caprifoliaceae	*Valeriana officinalis* L., SE123	palderjan	roots	tea	calming	1
				tincture, drunk	calming	2
					does not remember	1
					heart relaxation	1
Caryophyllaceae	*Stellaria media* (L.) Vill., SE069	naserm	aerial parts	fresh, topical application	inflammation (cyst)	1
Crassulaceae	*Phedimus stevenianus* (Rouy & E.G.Camus)‘t Hart	kuldjuur	roots	tincture, topical application	back pain	1
					joint pain	1
Cucurbitaceae	*Cucumis sativus* L.	kurk	fruits	finger inserted into fermented cucumber	abscesses	1
				topical application	for the eyes	1
Cupressaceae	*Juniperus communis* L., SE116	kadakas, kadaja, kattai	fruits	eaten	healthy	1
				eaten dried	to strengthen the immune system	1
				tincture	stomach pain	1
			twigs	applied honey on the body and whisked in sauna	back pain	2
					joint pain	1
				whisked in sauna	good for bones	2
					healthy	2
					itching	1
					joint pain	3
					nerve pain	1
					radiculitis	1
Elaeagnaceae	*Hippophae rhamnoides* L.	astelpaju	fruits	eaten frozen	vitamins	1
				tea	vitamins	1
Ericaceae	*Arctostaphylos uva-ursi* (L.) Spreng.	leesikas	aerial parts	tea	cold	3
			leaves	tea	bladder disease	5
					incontinence	1
					inflammation	1
	*Calluna vulgaris* (L.) Hull, SE093	kanarbik	aerial parts	tea	calming	1
					helpful for sleeping	1
	*Rhododendron tomentosum* Harmaja, SE038	sookail	aerial parts	tea	lung disease	1
	*Vaccinium myrtillus* L., SE021	mustk’, mustik’, mustikas, mustikad	fruits	eaten dried	stomach pain	3
					stomach problems	1
				eaten	diarrhoea	1
					healthy	2
					medicine	1
				eaten frozen	stomach problems	1
				eaten raw	diarrhoea	1
					good for the eyes	1
					stomach ache	2
					stomach pain in children	1
				jam eaten	diarrhoea	5
					diarrhoea in children	1
					good for the eyes	2
					stomach pain	4
					stomach problems	2
				juice drunk	diarrhoea	1
				macerated in vodka	sore throat	1
				tincture	stomach pain	1
			stems	tea	blood pressure	2
					medicine	1
	*Vaccinium oxycoccos* L., SE109	jõhvikas, kuremari	fruits	eaten raw	fever	2
					medicine	1
					high blood pressure	1
					vitamins	1
					good for blood circulation	1
					good for the heart	1
					healthy	1
				jam eaten	fever	1
				juice drunk	fever	2
				tea	fever in children	1
					healthy	1
	*Vaccinium vitis-idaea* L., SE127, SE035	pohl, palohkas	fruits	eaten fresh	fever	1
					healthy	1
				jam eaten	healthy	1
				juice drunk	inflammation	1
			leaves	tea	bladder disease	1
					bladder problems	1
					inflammation	1
					kidney cleansing	1
			stems	tea	bladder disease	1
					medicine	1
Fagaceae	*Quercus robur* L., SE100	tamm	bark	tea	diarrhoea	1
					stomach pain	1
					stomach problems	1
			twigs	whisked in sauna	healthy	1
Grossulariaceae	*Ribes nigrum* L., SE015	sitika, mustsõstar	fruits	jam eaten	fever	1
					sore throat	2
					stomach pain in children	1
					vitamins	1
				jam mixed in hot water	fever	1
					sore throat	1
				punch with vodka	cold	1
				raw	vitamin C	1
				tea	cold	1
					cold diseases	1
					fever	2
			leaves	tea	vitamins	1
			stems	tea	tonic	1
	*Ribes uva-crispa* L.	tikker, karusmari	fruits	eaten frozen	sore throat	1
Hypericaceae	*Hypericum perforatum* L., SE002, SE003, SEDR020, SEDR031	naistepuna	aerial parts	tea	100 diseases	1
	*Hypericum* sp., SE059, SEDR012, SEDR028	naistepuna	aerial parts	added to sauna whisk	prophylactics	1
				drunk with honey	cold	1
				tea	abdominal problems	1
					against nine diseases	1
					calming	1
					helpful for sleeping	1
					medicine	3
					prevention of women’s diseases	1
					women diseases	1
Lamiaceae	*Hyssopus officinalis* L.	iisop	aerial parts	tea	medicine	1
	*Mentha × piperita* L., SE025, SE064a, SE063b, SEDR014, SEDR035	must münt	aerial parts	tea	digestive problems	1
	*Mentha* sp., SE078, SE079, SEDR005, SEDR021, SEDR011	piparmünt, aiapiparmünt, mjäta	aerial parts	drunk with honey	cold	1
				tea	abdominal problems	1
					calming	4
					cold	4
					good for respiratory tract	2
					internal (abdominal) pains	1
					medicine	3
	*Mentha suaveolens* Ehrh., SE064b, SE060, SE075, SE065, SE063a, SE103	õunmünt	aerial parts	tea	digestive problems	1
	*Nepeta cataria* L., SE061, SE045, SEDR033	meliss	aerial parts	added to sauna whisk	prophylactics	1
				tea	calming	1
					gives a good night’s sleep	1
	*Origanum vulgare* L., SE082, SE090, SEDR032	pune	aerial parts	tea	medicine	3
	*Thymus pulegioides* L., SEDR016	nõmmeliivatee	aerial parts	tea	bronchitis	2
					cough	2
	*Thymus serpyllum* L., SE037, SEDR008	nõmm-liivatee, jaanihain, maarjahein	aerial parts	tea		
	
					cold	1
					cough	1
					medicine	3
					sore throat	1
	*Thymus* sp.	aed-liivatee	aerial parts	tea	calming	1
Leguminosae	*Trifolium pratense* L., SE085	punane ristik, ristik	flowers	tea	female disease (menstrual pain)	1
	*Trifolium repens* L., SE032	valge ristik	flowers	tea	medicine	1
Linaceae	*Linum usitatissimum* L.	lina	seeds	eaten soaked	stomach problems	1
				ointment, topical application	wounds	1
Malvaceae	*Tilia cordata* Mill., SE087, SE006, SEDR007, SEDR023, SEDR025, SEDR030, SEDR040	pähn, pärn, lõhmus	flowers	tea (with honey)	cold	9
					cough	6
					fever	13
					medicine	7
					sore throat	2
Myrtaceae	*Syzygium aromaticum* (L.) Merr. & L.M.Perry	nelk	seeds	topical application	toothache	2
Oleaceae	*Syringa vulgaris* L.	sirel	flowers	eaten dried	application not remembered	1
				tincture, topical application	joint pain	1
Onagraceae	*Epilobium angustifolium* L., SE049, SEDR034, SEDR036, SEDR037, SEDR045, SEDR047, SEDR048	põdrakanep, jaanihain, ivan-tšai	aerial parts	tea	for men to increase their potency	2
					inflammation	1
					strengthening the organism	1
			flowering tops	tea	improves immunity	1
					men’s Viagra	1
					prostate diseases	1
			flowers	tea	good for men	1
					inflammation	1
				tincture	medicine	1
			leaves	tea	improves immunity	1
					strengthens the body	1
Papaveraceae	*Chelidonium majus* L., SE039, SE050, SE098	vereurmarohi	Sap	juice, topical application	toenail fungus	2
Pinaceae	*Picea abies* (L.) H.Karst., SE126	kuusk	resin	fresh, topical application	wounds	8
				ointment (with lard), topical application	wounds	6
				ointment with goose fat, topical application	wounds	1
				tincture, topical application	wounds	1
				topical application	abscesses	3
					small wounds	3
			shoots	syrup	strengthens the organism	1
				tea	lung disease	1
				tincture	vitamins	1
			twigs	bathe with decoction	healthy	1
	*Pinus sylvestris* L., SE120	pedäja, pedaja, mänd	resin	ointment (with lard), topical application	wounds	6
					medicine	1
				fresh, topical application	wounds	3
			shoots	eaten fresh	strengthens the organism	1
				preserved in honey	vitamins	1
				tea	lung disease	1
				tincture, drunk	gastric diseases	1
					to clean blood vessels	1
				tincture, topical application	pimples on face	1
Piperaceae	*Piper nigrum* L.	pipar, must pipar	seeds	eaten	diarrhoea	2
					stomach ache	1
				taken with vodka	diarrhoea	3
					stomach ache	5
Plantaginaceae	*Plantago major* L., SE007, SE012	muroleht, murohain, paiseleht, muruhain, muruleht, teeleht, podorošnik, padarošnik, suur teeleht	leaves	eaten dried	stomach pain	1
				tea	expectorant	1
					medicine	2
				topical application	abscesses	2
					bleeding	8
					earache	1
					inflamed wounds	1
					inflammation	1
					painful legs	2
					painful areas	5
					rotting wounds	1
					scratches	6
					small wounds	1
					sore feet	1
					toothache	1
					wounds	15
Poaceae	*Avena sativa* L.	kaer	grains	warmed, topical application	sore throat	2
					back pain	1
Polygonaceae	*Rheum rhaponticum* L.	rabarber	leaves	sit on in sauna	healthy	4
Primulaceae	*Primula veris* L., SEDR003, SEDR039, SEDR042	kikkapüks, nurmenukk	flowers	tea	asthma	1
					cold	1
					good for the heart	1
					headache	1
					medicine	1
				tincture	medicine	1
Ranunculaceae	*Anemone nemorosa* L.	valge ülane	aerial parts	topical application	back pain	1
Rosaceae	*Alchemilla vulgaris* auct. (coll.), SE080	kortsleht	leaves	tea	after childbirth	1
					fever	1
					medicine	2
					women’s diseases	1
				topical application	bleeding	1
	*Aronia melanocarpa* (Michx.) Elliott, SE057, SE114	aroonia	fruits	juice drunk	high blood pressure	5
	*Chaenomeles japonica* (Thunb.) Lindl. ex Spach	ebaküdoonia	fruits	syrup	strengthens the organism	1
	*Filipendula ulmaria* (L.) Maxim., SE041, SEDR036, SEDR046	angervaks	flowers	tea	blood thickness	1
					calming	1
					fever	1
					medicine	1
					varicose veins	1
				tincture	medicine	1
				tincture, topical application	joint pain	1
	*Potentilla erecta* (L.), SE023	tedremaran	roots	tea	bleeding	1
				tincture	to promote childbirth	1
				topical application	bleeding	1
				washed with tea	toenail fungus	1
	*Prunus domestica* L.	ploom	fruits	juice drunk	constipation in children	1
	*Prunus padus* L., SE013	toomingas	fruits	dried	stomach pain	1
	*Rosa* sp., SE139	kibuvits	fruits	tea	cold	2
					healthy	1
					medicine	1
					vitamins	1
	*Rubus idaeus* L., SE027, SEDR029b	malina, vaarikad, vaarikas, vabarnad	fruits	jam eaten	fever	2
				tea	cold	3
				tea	fever	1
			leaves	tea	medicine	1
					tonic	1
			stems	tea (with honey)	cold	2
					cough	1
					fever	1
	*Rubus polonicus* Weston, SE040	tseavabarna, metsvabarna	stems	tea	cold	1
	*Sorbus aucuparia* L., SE026	pihlakas	fruits	compote eaten	source of vitamins	1
				eaten raw	vitamins	2
					to strengthen the immune system	1
Rubiaceae	*Coffea* sp.	kohv, must kohv, oakohv	seeds	eaten (with little water)	diarrhoea	5
Sapindaceae	*Aesculus hippocastanum* L., SE011, SEDR017	kastan, hobukastan, kaštan	flowers	tincture, topical application	back pain	1
					joint pain	2
			horse-chestnuts	kept in the pocket	disease prophylactics	1
				tincture, topical application	back pain	1
					joint pain	8
					varicose veins	1
Scrophulariaceae	*Verbascum thapsus* L.	üheksaväeline, seitsmeväeline	flowers	tincture	medicine	1
Solanaceae	*Capsicum annuum* L.	pipar, tšilli pipar, punane pipar, verikõder, mõru pipar	fruits	tincture	cough	1
					medicine	1
					stomach pain	2
				tincture, topical application	joint pain	2
	*Nicotiana rustica* L.	tubakas	leaves	inhaled powder	headache	1
	*Solanum lycopersicum* L.	tomat	fruits	eaten as salad	antiseptic	1
	*Solanum tuberosum* L.	kartul, (tärklis)	starch	eaten with little water	diarrhoea	3
			tubers	decocted, vapor inhaled	cold	1
					cough	4
					respiratory problems	1
					sore throat	1
				grated, topical application	eye inflammation	1
Theaceae	*Camellia sinensis* (L.) Kuntze	roheline tee	leaves	tea	back pain	1
					prophylactics	1
Urticaceae	*Urtica dioica* L., SE074, SEDR037, SEDR041	nõges, kõrvenõges, raudnõges	aerial parts	bathe with decoction	foot pain	1
				eaten in spring	blood cleansing	1
					strengthens the body	2
					vitamins in springtime	1
				tea	blood cleansing	1
					blood pressure	1
					medicine	2
					strengthens the body	2
					vitamins	2
				whisked in sauna	joint pain	4
					knee pain	1
					prophylactics	2
					radiculitis	2
					rheumatism	1
					back pain	2
			leaves	tea	healthy	1
					vitamins	1
			roots	boiled, foot bath	rheumatism	1
Zingiberaceae	*Zingiber officinale* Roscoe	ingver	roots	tea	cold	1

**Table 2:** Correspondence between emic disease names with etic disease categories and their frequency of mentioning.

**Blood: 7**	**Cardiovascular: 19**	**Male Genital: 4**
blood cleansing: 4	high blood pressure: 9	for men to increase their potency: 2
strengthen the blood: 2	blood pressure: 4	men’s Viagra: 1
blood thickness: 1	to clean blood vessels: 3	prostate diseases: 1
	varicose veins: 2	
	heart relaxation: 1	
**Neurological: 6**	**Pregnancy, Childbearing, Family Planning: 2**	**Eye: 3**
headache: 5	after childbirth: 1	eye inflammation: 1
nerve pain: 1	to promote childbirth: 1	for the eyes: 1
		painful eyes: 1
**Ear: 2**	**Endocrine: 1**	**Female Genital: 3**
earache: 2	diabetes: 1	women’s diseases: 2
		menstrual pain: 1
**Urological: 12**	**Psychological: 17**	**Musculoskeletal: 66**
bladder disease: 8	calming: 13	joint pain: 33
bladder problems: 1	helpful for sleep: 2	back pain: 14
incontinence: 1	gives a good night’s sleep: 1	knee pain: 5
kidney cleaning: 1	good sleep: 1	painful legs: 4
kidney disease: 1		radiculitis: 3
		rheumatism: 2
		muscle pain: 2
**Respiratory: 114**		
cold: 52	bronchitis: 3	cough in children: 2
cough: 30	lung disease: 3	respiratory problems: 1
sore throat: 17	expectorant: 2	cold diseases: 1
throat ache: 5	stuffy nose: 2	asthma: 1
**Digestive: 99**		
diarrhoea: 23	constipation in children: 2	indigestion: 1
stomach pain: 21	dental inflammation: 2	inflammation of the stomach: 1
stomach ache: 8	digestive problems: 2	internal (abdominal) pains: 1
toothache: 8	internal parasites: 2	liver cleansing: 1
stomach problems: 6	stomach upset: 2	oral inflammation: 1
stomach pain in children: 5	diarrhoea in children: 1	stomach gas in children: 1
gastric diseases: 4	gum disease: 1	stomach worms:
abdominal problems: 3	after stomach surgery: 1	strengthens the liver: 1
**Skin: 129**		
wounds: 45	burns: 3	hair loss: 1
abscesses 23	toenail fungus: 3	insect bites: 1
Bleeding: 14	topic swelling: 3	psoriasis: 1
painful areas: 11	inflamed wounds: 2	rotting wounds: 1
scratches: 7	scabies: 2	skin diseases: 1
pimples on face: 4	bee stings: 1	sore feet: 1
small wounds: 4	cuts: 1	
**General: 249**		
medicine 71	fever in children: 2	good for blood vessels: 1
fever: 33	good for bones: 2	good for every sickness: 1
inflammation: 25	good for everything: 2	good for the eyes: 1
healthy: 24	good for health: 2	good for men: 1
vitamins: 13	good for the heart: 2	good for the stomach: 1
strengthen the immune system: 7	good for the respiratory tract: 2	good medicinal herb: 1
prophylactics: 5	improves immunity: 2	inflammation (cyst): 1
vitamins in springtime: 5	strengthening the organism: 2	inflammation (?): 1
good for the eyes: 4	tonic: 2	itching: 1
prophylactic for worms: 4	unknown: 2	prevention of women’s disease: 1
strengthens the body: 4	disease prophylactics: 1	prophylactic for disease: 1
antiseptic: 3	disease prevention: 1	several diseases: 1
strengthens the organism: 3	don’t know exactly: 1	sick: 1
100 diseases: 2	feeling bad: 1	swollen legs: 1
against 9 diseases: 2	for strengthening the body: 1	vitamin C: 1
does not remember: 2	good for blood circulation: 1	vitamins in wintertime: 1

**Table 3:** Overlap in the used plant taxa in historical sources and the current study.

	Historical	Current
JI for taxa with at least three UR as a sum	**64.1**	**85.45**
JI for taxa with at least three UR in each group	47.17	52.08
JI for all identified taxa	47	54.74
JI with unidentified folk taxa	41.23	-
No. of taxa Setos (+unidentified)	73 + 9	67
No. of taxa Võros (+unidentified)	75 + 5	69

**Table 4:** Taxa having at least three UR and not mentioned in more than two UR in historical sources. Columns in green: data reflecting plant use in 19th and 20th century on the territory of current Estonia. *—different use, &—use provided by a healer, most likely relying on books.

Region/Period (If Not Current)	Latgale, Latvia	Ljuban, Belarus	Bukovina, Ukraine	Bukovina, Ukraine	Bukovina, Ukraine	Estonia/ 1930s	Estonia/ 1890s	Pernau, Livonia/1830s
Taxa/source	[43]	[44,45]	[46]	[41]	[42]	[48]	[49]	[47]
*Aesculus hippocastanum*	X	X	X		X			
*Alchemilla vulgaris*	X					X *	X	
*Allium sativum*	X	X	X	X	X			
*Armoracia rusticana*	X	X	X	X	X		X*	
*Aronia melanocarpa*	X	X	X					
*Brassica oleraceae*	X	X	X	X	X		X*	
*Calendula officinalis*	X	X	X	X	X			
*Coffea*								
*Echinacea purpurea*								
*Epilobium angustifolium*	X	X		X		X *&		X *
*Filipendula ulmaria*	X					X *&		X
*Hypericum*	X	X	X	X	X	X&		X
*Nepeta cataria*	X	X						
*Origanum vulgare*	X	X	X	X	X	X &		X
*Piper nigrum*	X	X						
*Primula veris*	X		X	X	X	X &		
*Rheum rhaponticum*								
*Ribes nigrum*	X	X	X	X				
*Rosa*	X	X	X	X		X *&		
*Sinapis alba*	X							
*Taraxacum officinale*	X	X	X	X	X	X &		
*Thymus pulegioides*								
*Tussilago farfara*	X	X	X	X	X	X &	X	
*Vaccinium oxycoccos*	X	X						

## Data Availability

All the data will be made publicly available after the end of the project.

## References

[1] Pardo-de-Santayana M., Quave C.L., Sõukand R., Pieroni A., Heinrich M., Jäger A.K. (2015). Medical ethnobotany and ethnopharmacology of Europe. Ethnopharmacology.

[2] Pieroni A., Vandebroek I., Prakofjewa J., Bussmann R.W., Paniagua-Zambrana N.Y., Maroyi A., Torri L., Zocchi D.M., Dam A.T.K., Khan S.M. (2020). Taming the pandemic? The importance of homemade plant-based foods and beverages as community responses to COVID-19. J. Ethnobiol. Ethnomed..

[3] Menendez-Baceta G., Aceituno-Mata L., Molina M., Reyes-García V., Tardío J., Pardo-de-Santayana M. (2014). Medicinal plants traditionally used in the northwest of the Basque Country (Biscay and Alava), Iberian Peninsula. J. Ethnopharmacol..

[4] Jernigan K.A., Belichenko O.S., Kolosova V.B., Orr D.J. (2017). Naukan ethnobotany in post-Soviet times: Lost edibles and new medicinals. J. Ethnobiol. Ethnomed..

[5] Quave C.L., Pieroni A. (2015). A reservoir of ethnobotanical knowledge informs resilient food security and health strategies in the Balkans. Nat. Plants.

[6] Medeiros P.M.D., Soldati G.T., Alencar N.L., Vandebroek I., Pieroni A., Hanazaki N., de Albuquerque U.P. (2012). The use of medicinal plants by migrant people: Adaptation, maintenance, and replacement. Evid. Based Complement. Altern. Med..

[7] Menendez-Baceta G., Aceituno-Mata L., Reyes-García V., Tardío J., Salpeteur M., Pardo-de-Santayana M. (2015). The importance of cultural factors in the distribution of medicinal plant knowledge: A case study in four Basque regions. J. Ethnopharmacol..

[8] Pieroni A., Sõukand R., Bussmann R.W. (2020). The Inextricable Link Between Food and Linguistic Diversity: Wild Food Plants among Diverse Minorities in Northeast Georgia, Caucasus. Econ. Bot..

[9] Pieroni A., Sõukand R. (2018). Forest as stronghold of local ecological practice: Currently used wild food plants in Polesia, Northern Ukraine. Econ. Bot..

[10] Kalle R., Sõukand R., Pieroni A. (2020). Devil is in the details: Use of wild food plants in historical Võromaa and Setomaa, present-day Estonia. Foods.

[11] Palo A., Külvik M., Palo K., Puura I. (2016). Taimkate. Setomaa.

[12] Kull T., Külvik M., Palo K., Puura I. (2016). Taimestik. Setomaa.

[13] Kukk Ü., Külvik M., Palo K., Puura I. (2016). Taimestik ja selle haruldused. Setomaa.

[14] Jääts I. (2000). Ethnic identity of the Setus and the Estonian-Russian border dispute. Natl. Pap..

[15] Kalkun A. (2019). Seto kultuur: Eksootiline võõras ja lähedane oma. Horisont.

[16] Laid E. (1935). Setumaa eestistub kiiresti. Postimees.

[17] (1935). Ringi ümber kodumaa. Postimees.

[18] (1936). Ringi ümber kodumaa. Postimees.

[19] (1937). Ringi ümber kodumaa. Postimees.

[20] Korb A., Kalkun A. (2007). Setod Siberimaal. Sirp.

[21] (1936). Setud igatsevad Läti lihapotte. Postimees.

[22] (1936). Ringi ümber kodumaa. Postimees.

[23] (1938). Ringi ümber kodumaa. Postimees.

[24] (1940). Läbi kodumaa linnade. Postimees.

[25] Assmuth L., Berglund J., Lundén T., Strandbrink P. (2015). Intertwining identities: The politics of language and nationality in the Estonian-Russian borderlands. Crossings and Crosses: Borders, Educations, and Religions in Northern Europe.

[26] Lüüs A. (1960). Rahvahaigused ja rahva ravimisviisid Võrumaal 19. sajandi viimasel veerandil. II. Mana.

[27] Lüüs A. (1959). Rahvahaigused ja rahva ravimisviisid Võrumaal 19. sajandi viimasel veerandil. I. Mana.

[28] Gustavson H., Pilk F.R. (1982). Kreutzwaldi arstitegevusse. Keel Kirjand..

[29] Gustavson H. (2020). Eesti Apteekide Ajaloost.

[30] Field M.G., Cooter R., Pickstone J. (2020). Soviet medicine. Medicine in the Twentieth Century.

[31] Schecter K. (1992). Soviet socialized medicine and the right to health care in a changing Soviet Union. Hum. Rights Q..

[32] Barr D.A. (1996). The ethics of Soviet medical practice: Behaviours and attitudes of physicians in Soviet Estonia. J. Med. Ethics.

[33] OECD State of Health in the EU Estonia: Country Health Profile 2019. https://ec.europa.eu/health/sites/default/files/state/docs/2019_chp_et_english.pdf.

[34] Assmuth L., Bacas J.L., Kavanagh W. (2013). Asymmetries of gender and generation in a post-Soviet borderland. Border Encounters. Asymmetry and Proximity at Europe’s Frontiers.

[35] International Society of Ethnobiology International Society of Ethnobiology Code of Ethics (with 2008 Additions). http://ethnobiology.net/code-of-ethics/.

[36] Plants of the World Online Facilitated by the Royal Botanic Gardens, Kew, UK. https://powo.science.kew.org/.

[37] Tutin T.G., Burges N.A., Chater A.O., Edmondson J.R., Heywood V.H., Moore D.M., Valentine D.H., Walters S.M., Webb D.A. (1964–1980). Flora Europaea.

[38] Stevens P.F. Angiosperm Phylogeny Website. 2001 Onwards. Version 14, July 2017. http://www.mobot.org/MOBOT/research/APweb/.

[39] González-Tejero M., Casares-Porcel M., Sánchez-Rojas C.P., Ramiro-Gutiérrez J.M., Molero-Mesa J., Pieroni A., Giusti M.E., Censorii C., de Pasquale C., Della D. (2008). Medicinal plants in the Mediterranean area: Synthesis of the results of the project Rubia. J. Ethnopharmacol..

[40] Sõukand R., Kalle R. (2008). HERBA: Historistlik Eesti Rahvameditsiini Botaaniline Andmebaas.

[41] Mattalia G., Stryamets N., Pieroni A., Sõukand R. (2020). Knowledge transmission patterns at the border: Ethnobotany of Hutsuls living in the Carpathian Mountains of Bukovina (SW Ukraine and NE Romania). J. Ethnobiol. Ethnomed..

[42] Mattalia G., Stryamets N., Grygorovych A., Pieroni A., Sõukand R. (2021). Borders as crossroads: The diverging routes of herbal knowledge of Romanians living on the Romanian and Ukrainian sides of Bukovina. Front. Pharmacol..

[43] Simanova A., Prūse B., Kalle R., Kochalski S., Prakofjewa J., Mežaka I., Pieroni A., Krūzkopa S., Holsta I., Sõukand R. (2020). Medicinal plant use at the beginning of the 21st century among the religious minority in Latgale Region, Latvia. Ethnobot. Res. Appl..

[44] Sõukand R., Hrynevich Y., Vasilyeva I., Prakofjewa J., Vnukovich Y., Paciupa J., Hlushko A., Knureva Y., Litvinava Y., Vyskvarka S. (2017). Multi-functionality of the few: Current and past uses of wild plants for food and healing in Lubań region, Belarus. J. Ethnobiol. Ethnomed..

[45] Sõukand R., Hrynevich Y., Prakofjewa J., Valodzina T., Vasilyeva I., Paciupa J., Shrubok A., Hlushko A., Knureva Y., Litvinava Y. (2017). Use of cultivated plants and non-plant remedies for human and animal home-medication in Liubań district, Belarus. J. Ethnobiol. Ethnomed..

[46] Sõukand R., Pieroni A. (2016). The importance of a border: Medical, veterinary, and wild food ethnobotany of the Hutsuls living on the Romanian and Ukrainian sides of Bukovina. J. Ethnopharmacol..

[47] Kalle R., Sõukand R. (2021). The name to remember: Flexibility and contextuality of preliterate folk plant categorization from the 1830s, in Pernau, Livonia, historical region on the eastern coast of the Baltic Sea. J. Ethnopharmacol..

[48] Sõukand R., Kalle R. (2012). Personal and shared: The reach of different herbal landscapes. Est. J. Ecol..

[49] Kalle R., Pieroni A., Svanberg I., Sõukand I. (2022). Early Citizen Science Action in Ethnobotany: The Case of the Folk Medicine Collection of Dr. Mihkel Ostrov in the Territory of Present-Day Estonia, 1891–1893. Plants.

[50] Valk H., Aleksandrov A., Benfoughal T., Fišman O., Valk H. (2017). Setomaa pühad ja kultuslikud kivid. Inimese Muuseumi ekspeditsioonid Eestisse: Boris Vilde ja Leonid Zurov Setomaal (1937–1938).

[51] Raudoja A., Valk H., Aleksandrov A., Benfoughal T., Fišman O., Valk H. (2017). Setomaa pühapuud. Inimese Muuseumi ekspeditsioonid Eestisse: Boris Vilde ja Leonid Zurov Setomaal (1937–1938).

[52] Valk A., Grušina L., Benfoughal T., Fišman O., Valk H. (2017). Setomaa pühad allikad ja veekogud. Inimese Muuseumi ekspeditsioonid Eestisse: Boris Vilde ja Leonid Zurov Setomaal (1937–1938).

[53] Guarrera P.M. (2006). Usi e Tradizioni della Flora Italiana. Medicina popolare ed etnobotanica.

[54] Sõukand R., Mattalia G., Kolosova V., Stryamets N., Prakofjewa J., Belichenko O., Kuznetsova N., Minuzzi S., Keedus L., Prūse B. (2020). Inventing a herbal tradition: The complex roots of the current popularity of Epilobium angustifolium in Eastern Europe. J. Ethnopharmacol..

[55] Sõukand R., Kalle R., Svanberg I. (2010). Uninvited guests: Traditional insect repellents in Estonia used against the clothes moth *Tineola bisselliella*, human flea *Pulex irritans* and bedbug *Cimex lectularius*. J. Insect Sci..

